# Exploring the potential of zinc-solubilizing *Bacillus* strains to enhance rice (*Oryza sativa* L.) productivity in nutrient-deficit soils

**DOI:** 10.3389/fmicb.2025.1626216

**Published:** 2025-09-03

**Authors:** Maqshoof Ahmad, Iqra Naseer, Farheen Nazli, Abubakar Dar, Rubab Sarfraz, Usman Zulfiqar, Hossam S. El-Beltagi, Muneera A. Saleh, Nazih Y. Rebouh, Xasanboy Rasulov, PV Vara Prasad

**Affiliations:** ^1^Department of Soil Science, The Islamia University of Bahawalpur, Bahawalpur, Pakistan; ^2^Institute of Agro-Industry and Environment, The Islamia University of Bahawalpur, Bahawalpur, Pakistan; ^3^Institute of Agriculture and Applied Life Science, Gyeongsang National University, Jinju, Republic of Korea; ^4^Department of Agronomy, Faculty of Agriculture and Environment, The Islamia University of Bahawalpur, Bahawalpur, Pakistan; ^5^Department of Agricultural Biotechnology, College of Agriculture and Food Sciences, King Faisal University, Al-Ahsa, Saudi Arabia; ^6^Department of Biology, College of Sciences, Taif University, Taif, Saudi Arabia; ^7^Department of Environmental Management, Institute of Environmental Engineering, RUDN University, Moscow, Russia; ^8^Department of Agrochemistry and Soil Science, Tashkent State Agrarian University, Tashkent, Uzbekistan; ^9^Department of Agronomy, Kansas State University, Manhattan, KS, United States

**Keywords:** *Bacillus*, malnutrition, quality, micronutrients, soil health, rice

## Abstract

**Introduction:**

Food and nutritional security remain a significant challenge among the food-insecure people around the world, facing a lack of nutritious food rather than food availability alone. Micronutrient deficiencies in staple grains present a serious public health issue, especially impacting millions of women and children in developing nations. Staple cereals contain low concentrations of micronutrients, especially zinc (Zn).

**Methodology:**

The present study explored the potential of zinc-solubilizing *Bacillus* strains to improve rice (*Oryza sativa* L.) growth, antioxidant activity, yield, and quality in a completely randomized design (CRD) with four replications. For this purpose, four pre-isolated, characterized and identified *Bacillus* strains (AN24, AN30, AN31, and AN35) were evaluated separately, as well as in co-inoculation on the growth promotion of rice cultivar PK 386.

**Results:**

The results showed that the co-inoculation of *Bacillus* strains improved the growth and yield of rice more effectively than individual bacterial strains. Furthermore, co-inoculation was also more efficient in improving the soil nutrient status and biology (microbial populations) on which rice plants were grown in the pot experiment. In addition to improvement in plant growth parameters, the co-inoculation of *Bacillus* strains improved the N, P, K, Fe, and Zn up to 26, 30, 29, 19, and 27%, respectively, in rice grains as compared to control, along with improvement in macro and micronutrients in rice straw and roots. Co-inoculation also improved the crude protein in rice grains by 27% compared to the un-inoculated control.

**Discussion:**

These results suggest that co-inoculated *Bacillus megaterium* strains AN24 and B. AN31offer a promising, eco-friendly alternative to synthetic fertilizers and can play a vital role in addressing micronutrient deficiencies in cereals.

**Future directions:**

Further molecular characterization of Zn solubilizing genes and field-scale evaluations are recommended to validate their efficacy under diverse agroecological conditions. The combination could be further evaluated as a valuable tool for developing biofertilizers to improve rice productivity and quality in nutrient-deficient soils.

## 1 Introduction

The global human population has been increasing rapidly, particularly during the past two decades, which puts pressure on the food systems (Rahut et al., 2022). Therefore, food insecurity under the prevailing climate change scenario is one of the significant challenges the modern world faces (Lopes et al., 2023; Zulfiqar et al., 2024). At present the food-insecure people face the issue of low-quality food rather than its availability. Micronutrient deficiency in food grains thus poses the most serious health threat, affecting millions of women and children, particularly in developing countries (Kiran et al., 2022). Staple cereals contain low concentrations of micronutrients, especially zinc (Zn) and iron (Fe) (Hotegni et al., 2024).

Rice (*Oryza sativa* L.) is the second most important staple food grain consumed by about half of the world’s population (Mohidem et al., 2022). It is cultivated globally in over a hundred countries (Mahmood et al., 2023) and Asia alone contributes about 90% of the total rice production (Fukagawa and Ziska, 2019). Rice is a significant source of carbohydrates and provides other nutrients essential for human beings, including minerals, folate, niacin, and vitamins (Zafar and Xu, 2023). Zinc, the essential micronutrient, is crucial for several physiological and biochemical processes in rice plants, such as membrane integrity, redox reactions, enzyme activities and chlorophyll synthesis (Saleem et al., 2022). Deficiency of Zn in plants disrupts important metabolic functions, leading to stunted growth and leaf chlorosis (Suganya et al., 2020). Lower Zn in grains contributes to Zn deficiency in the human diet (Saleem et al., 2022).

Arid and semi-arid region soils are often low in organic matter and essential plant nutrients which are being more degraded due to intensive farming (Quoreshi et al., 2022). Alkaline and calcareous soil with inherently low organic matter, salinity, and waterlogging issues are more prone to Zn deficiency (El-Beltagi et al., 2022a; Martínez-Ríos et al., 2024). These soils, however, can be productive for growing crops through effective soil fertility management strategies (Shah and Wu, 2019; Srivastava et al., 2024). Any strategy that can help enhance the Zn uptake from soil and its translocation to the grain can help mitigate the issue of Zn deficiency (Kamran et al., 2023).

Several strategies are used to fortify cereal grains with micronutrients, including Zn and Fe. These approaches include conventional breeding approaches to develop varieties with higher nutrient contents, transgenic approaches to modify genetic makeup with a natural ability to produce fortified grains, industrial food fortification, and agronomic approaches (Naik et al., 2024). The calcareous and alkaline soils of Pakistan are the major reason behind low indigenous Zn availability and low Zn fertilizer use efficiency (FUE) due to Zn fixation in soil (Nadeem et al., 2024). So, total Zn concentration is not the issue in these soils under arid and semi-arid regions, but low Zn availability hinders the yield and quality of the produce (Saboor et al., 2021). The indigenous insoluble Zn compounds can be solubilized by Zn solubilizing bacteria (ZSB) (Yadav et al., 2022). These bacteria use different processes to solubilize the insoluble Zn compounds viz., proton extrusion, redox reactions, siderophores, exopolysaccharides (EPS) and organic acids production. The more studied mechanism is the production of low molecular weight organic acids (Mumtaz et al., 2017), which reduce pH in the microenvironment, thus inducing a favorable environment for Zn solubility.

Using ZSB to improve cereal grains’ crop productivity and nutrient quality under nutrient- deficit soil conditions is an eco-friendly, low-cost, and sustainable approach, as reported in the literature (Singh et al., 2024). Several strains of ZSB, characterized by their ability to solubilize unavailable Zn compounds to improve crop plants’ growth, yield, and grain quality (Mumtaz et al., 2017; Singh et al., 2024). Among these, strains of the genus *Bacillus* are widely distributed and reported in the literature to possess multifarious plant growth-promoting (PGP) traits (Mumtaz et al., 2017; Yadav et al., 2022). Using ZSB to improve cereals’ growth, yield, and quality is a widely accepted approach that can help enhance Zn concentration in grains, thus enriching the human diet with zinc (Aloo et al., 2022). The present study explored the potential of four ZSB *Bacillus* strains (AN24, AN30, AN31, and AN35) separately and in combination on growth, antioxidant activity, yield, and quality of rice.

## 2 Materials and methods

A pot experiment was conducted to explore the potential of ZSB strains to enhance the growth, antioxidant activity, yield, and quality of rice (*Oryza sativa* L.). For this purpose, four pre-isolated, characterized, and identified bacterial strains, *Bacillus megaterium* AN24, *B. aryabhattai* AN30, *B. megaterium* AN31, and *B. megaterium* AN35, were evaluated separately, and in co-inoculation for their potential to enhance the growth and yield of rice variety PK 386.

### 2.1 Collection of rice seeds and confirmation of their germination

Rice seeds of a variety widely accepted by the farming community, PK 386 was used for this research. The seeds were screened for husk or stones and surface sterilized using a 2% sodium hypochlorite solution. Fifty sterilized seeds with uniform size and high vigor were sown between two layers of Whatman filter paper and soaked in sterilized distilled water. The Petri dish was covered with a lid and incubated in the dark at 32 ± 1 °C. The germination was monitored, and the germination percentage was calculated as the ratio of germinated seeds and total number of seeds.


G⁢e⁢r⁢m⁢i⁢n⁢a⁢t⁢i⁢o⁢n%⁢a⁢g⁢e=N⁢o.o⁢f⁢s⁢e⁢e⁢d⁢s⁢g⁢e⁢r⁢m⁢i⁢n⁢a⁢t⁢e⁢dT⁢o⁢t⁢a⁢l⁢n⁢u⁢m⁢b⁢e⁢r⁢o⁢f⁢s⁢e⁢e⁢d⁢s⁢X⁢ 100

### 2.2 Nursery preparation and maintenance

For the preparation of the rice nursery, good-quality seeds with more than 90% germination rate were soaked in clean water for 24 h before sowing. After soaking, water was drained, and the seeds were transferred to a germination tray filled with a mixture of sieved soil and peat, covering them properly with a germination medium. Trays were placed in partially shaded, well-ventilated areas to provide proper light and temperature conditions. A good moisture level was maintained by regular irrigations.

### 2.3 Collection of *Bacillus* strains and preparation of inoculum

The zinc solubilizing *Bacillus* strains with siderophores and exopolysaccharides production ability viz. *Bacillus megaterium* AN24 (MN005926), *B. aryabhattai* AN30 (MN005927), *B. megaterium* AN31 (MN005928), and *B. megaterium* AN35 (MN005929) were collected from the Soil Microbiology and Biotechnology Laboratory, Department of Soil Science, ISWR, the Islamia University of Bahawalpur. These strains have also been documented for phosphorus (P) solubilization, catalase activity, chitinase activity, and protease activity, as well as the production of low-molecular-weight organic acids (Naseer et al., 2020).

The inoculum of the Zn solubilizing *Bacillus* strains was prepared using 0.85% saline solution (sterilized) following the method of Ramesh et al. (2014) with slight modifications (Singh et al., 2016) with 0.1% zinc oxide as zinc source. After inoculating the sterilized broth, the flasks were incubated in a shaking incubator (S19R-2, Sheldon Manufacturing, Cornelius, OR, USA) at 30 ± 1 °C for 72 h. A uniform population of inoculum (OD600 = 0.5 ∼ 106 to 108 CFU mL^–1^) was maintained using distilled water, and the same was used to inoculate rice seedlings before transplantation.

### 2.4 Experimentation

The experiment was conducted in a wire house under natural conditions, except the damage to animals and birds was controlled. The physiochemical analyses of the soil used for the pot experiment were performed following the standard protocols (Ryan et al., 2001) before filling the pots. The soil was normal (ECe = 1.4 dS m^–1^) with an alkaline reaction (pH = 8.0), having a sandy loam texture and a 38% saturation percentage. Moreover, it was deficient in nitrogen (N) (0.287 mg kg^–1^), P (5.38 mg kg^–1^), and zinc (0.68 mg kg^–1^) while adequate in potassium (K) (149 mg kg^–1^) with 0.58% organic matter. The clean soil was prepared adequately by removing stones and leaves and was used to fill pots (12 kg pot^–1^) and placed in the wire house from July to November 2023, with an average day length between 12–14 h and a 10–12 h night period. The temperature fluctuates between 35–45 ± 1 °C in the day and 25–30 ± 1 °C during the night. Thirty-days-old rice nursery was uprooted and inoculated with respective *Bacillus* strains (100 mL with OD600 = 0.5 ∼ 106 to 108 CFU mL^–1^, separately and in co-inoculation, by dipping the roots in inoculum for 20 min. For co-inoculation, the broth of respective *Bacillus* strains was mixed equally (1:1 ratio) 50 mL each while the control plants were dipped in sterilized broth for 20 min. The inoculated seedlings were transplanted into six holes (two plants per hole) in each pot and then thinned to three plants after 10 days to maintain a uniform plant population. The recommended doses of N (147 kg ha^–1^), P (86 kg ha^–1^), and K (62 kg ha^–1^) were applied as chemical fertilizers. The N and K were applied as basal doses, with the N split into three equal doses. Four replications (pots) for each treatment (T_1_ = Control, T_2_ = AN24, T_3_ = AN30, T_4_ = AN31, T_5_ = AN35, T_6_ = AN24–AN30, T_7_ = AN24–AN31, T_8_ = AN24–AN35, T_9_ = AN30–AN31, T_10_ = AN30–AN35 and T_11_ = AN31–AN35) were arranged in a completely randomized design (CRD) with pots randomization every fortnight for removal of experimental error. Good quality water (1000 mL per irrigation) was used for irrigation when required (Ayers and Westcot, 1985). Data on growth and yield parameters were measured at final harvest at physiological maturity. The root, straw, and grain samples were preserved to analyze the mineral contents in plants and grains. The soil samples were also collected and preserved to analyze soil mineral contents and biological attributes.

### 2.5 Plant analysis

The leaf chlorophyll index as were measured as SPAD value according to the standard method described by Ling et al. (2011). For this purpose, the SPAD meter (Model: SPAD-502, Minolta, Minolta Co., Ltd., UK) was used to take the values of the youngest mature leaf from each pot. The relative water content (RWC) in rice leaves were measured by collecting samples of the four fully opened, youngest leaves from each treatment. The fresh, fully turgid, and dry weight of selected leaves was recorded for calculating RWC (Mayak et al., 2004) as the ratio of difference between fresh weight and dry weight and fully turgid weight and dry weight, expressed as percentage.


Relative⁢Water⁢Content⁢(RWC)=

F⁢r⁢e⁢s⁢h⁢w⁢e⁢i⁢g⁢h⁢t-D⁢r⁢y⁢w⁢e⁢i⁢g⁢h⁢tF⁢u⁢l⁢l⁢y⁢t⁢u⁢r⁢g⁢i⁢d⁢w⁢e⁢i⁢g⁢h⁢t-D⁢r⁢y⁢w⁢e⁢i⁢g⁢h⁢t⁢X⁢ 100

To measure antioxidant enzyme activities, a 0.5 g properly ground leaf sample was homogenized with pre-cooled phosphate buffer (pH = 7.8). The contents were then centrifuged at 13000 rpm for 15 min using a refrigerated centrifuge adjusted at 4 °C. The supernatant was collected and preserved at 4 °C. The superoxide dismutase (SOD), peroxidase (POD), catalase (CAT), and ascorbate peroxidase (APX) activities in rice leaves were measured by following the methods of Beauchamp and Fridovich (1971), Gorin and Heidema (1976), Teranishi et al. (1974), and Asada and Takahashi (1987), respectively, with slight modifications.

Samples from rice grain, straw, and roots were digested using Wolf’s method (Wolf, 1982). The N contents in digested samples were measured by following the Kjeldahl method (Ryan et al., 2001), and the values were multiplied with a factor of 6.25 to get crude protein in rice grain (Thimmaiah, 2004). To analyze P in rice grain, straw, and roots, the samples were prepared according to the method of Chapman and Pratt (1961), and analysis was carried out on a UV-visible spectrophotometer (Carry 60, Made: Agilent Technologies, USA). The K contents were measured in digested plant samples using a Flame Photometer (Ryan et al., 2001), while Miller’s method was used to analyze Fe and Zn contents in digested samples (Miller, 1998) using an atomic absorption spectrophotometer (240FS, Agilent Technologies, USA).

### 2.6 Post-harvesting soil analysis

Post-harvesting soil was analyzed for important soil mineral contents and biological properties to study the impact of *Bacillus* strains on soil health. For this purpose, the soil was analyzed for organic matter contents (Moodie et al., 1959). Ginning and Hibbard’s H2SO4 digestion and distillation method was followed to analyze total N contents in soil, and the analysis was performed on the Kjeldahl apparatus (Jackson, 1962). Olsen’s method was used to analyze the available P content in soil (Watanabe and Olsen, 1965). The analysis for extractable K in soil was performed by extraction through ammonium acetate. The K contents in the filtrate were measured using a flame photometer (BWB XP, BWB Technologies, UK). The DTPA extraction method was followed to determine zinc and iron contents in post-harvest soil (Estefan et al., 2013). The culturable soil bacteria were enumerated using the dilution plate technique (cfu g^–1^ soil) on glucose peptone agar medium (GPM) following the method of Alexander (1982).

### 2.7 Statistical analysis

The data for the parameters studied were statistically analyzed using a one-way analysis of variance (ANOVA) under a completely randomized design (CRD) at a 5% significance level, following the method described by Steel et al. (1997). Treatment means and standard errors were calculated using Microsoft Excel (MS Office 2010 and 365). Where ANOVA results were significant, mean comparisons were performed using the least significant difference (LSD) test at the 5% probability level. Additionally, Pearson’s correlation plots and principal component analysis (PCA) biplots were generated using Origin Pro version 2024b (OriginLab Corporation, Northampton, USA).

## 3 Results

Seeds of rice variety PK 386 were collected from the local market and confirmed that they have over 90% germination by conducting a germination test in a Petri dish. Then, the ZSB strains were evaluated separately and in co-inoculation for their potential to enhance rice growth, physiology, yield, and quality. The details of the results are as follows.

### 3.1 Effect of ZSB strains on growth, yield, and yield contributing parameters of rice

**FIGURE 1:**
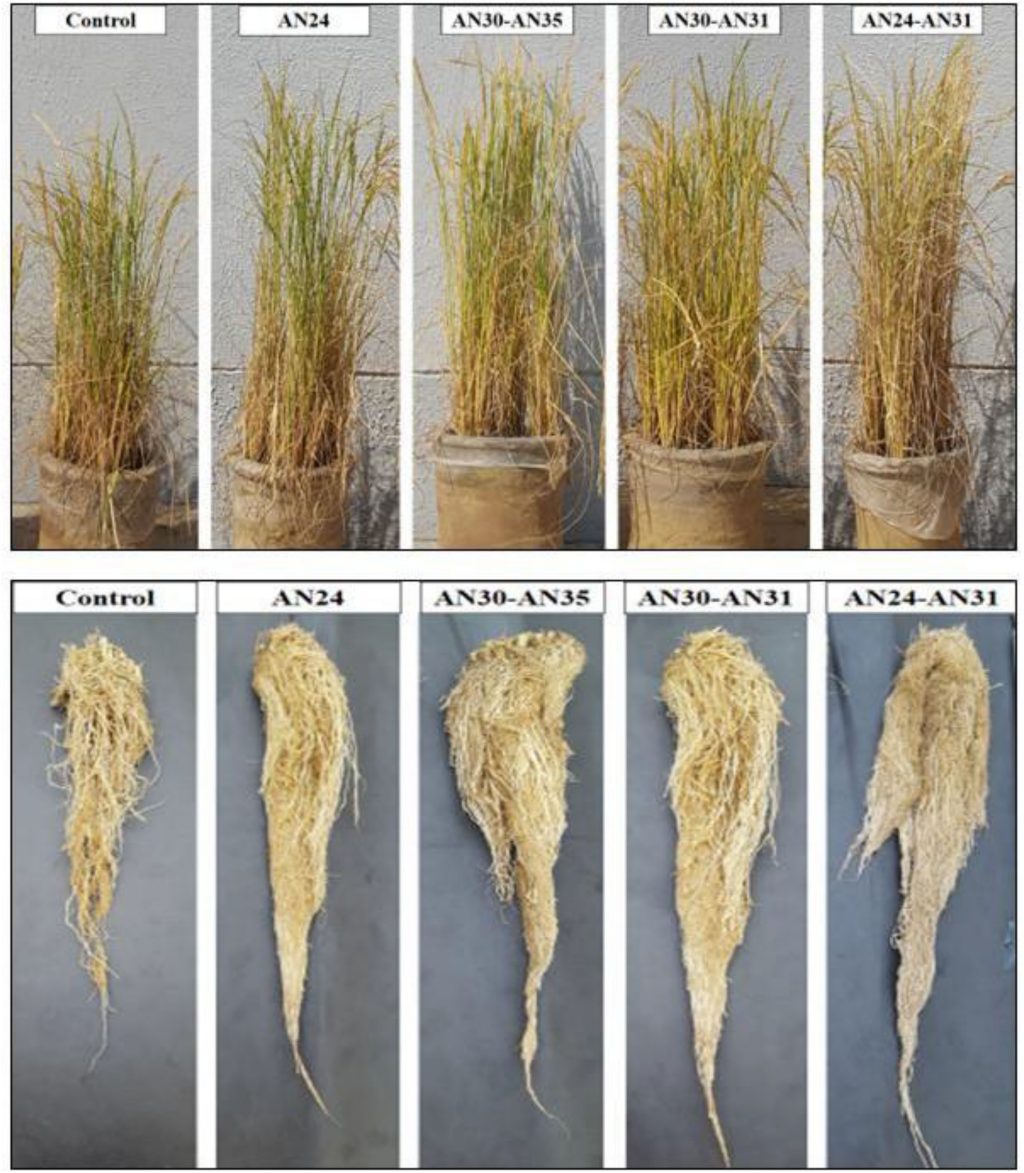
Efficacy of single and co-inoculation of zinc-solubilizing *Bacillus* strains in improving shoot and root growth of rice in pot experiment.

The ZSB strains significantly improved growth and yield parameters (Table 1). The sole inoculation of *Bacillus* strains significantly improved the plant height (8.83%–12.21%), and root length (10.42%–15.33%) compared to the un-inoculated control. However, they were statistically non-significant when compared with each other (*p* ≥ 0.05). The plant height and root length increase due to co-inoculation ranged from 5.19% to 15.32% and 17.05% to 22.74%, respectively, compared with the un-inoculated control. The maximum increase in plant height (15.32%) and root length (22.74%) was observed due to the combination of *B. megaterium* AN24 and *B. megaterium* sAN31, followed by the combination of *B. aryabhattai* AN30 and *B. megaterium* AN31. Visual (Figure 1) shows the effectiveness of co-inoculation treatment combinations of *B. aryabhattai* AN30 with *B. megaterium* AN31 and *B. megaterium* AN24 with *B. megaterium* sAN31 when compared to un-inoculated control.

**TABLE 1:** Effect single and co-inoculation of zinc-solubilizing *Bacillus* strains on growth parameters of rice (*n* = 4).

Treatment	Plant height (cm)	Root length (cm)	Root/ shoot ratio	Shoot dry biomass (g pot^–1^)	Root dry biomass (g pot^–1^)
Control	96.3 g	29.0 f	0.302 d	28.6 g	14.9 h
AN24	108.0 a-c	33.5 a-d	0.310 b-d	33.3 b-d	17.5 bc
AN30	104.8 c-f	32.5 c-e	0.310 b-d	31.9 def	16.8 def
AN31	106.0 b-e	32.1 c-e	0.302 d	32.4 c-f	17.1 c-e
AN35	107.0 a-d	33.1 b-e	0.309 b-d	32.9 b-e	17.3 b-d
AN24-AN30	102.5 ef	31.0 ef	0.302 d	30.8 f	16.3 fg
AN24-AN31	111.0 a	35.6 a	0.321 a	35.1 a	18.5 a
AN24-AN35	101.3 f	31.3 e	0.309 b-d	30.9 f	16.1 g
AN30-AN31	110.0 ab	34.9ab	0.317 ab	34.5 ab	18.2 a
AN30-AN35	108.8 a-c	34.0 a-c	0.312 a-c	33.9 a-c	17.9 ab
AN31-AN35	103.8 d-f	31.6 de	0.304 cd	31.5 ef	16.5 e-g
LSD value	4.2110	2.1723	0.0095	1.7777	0.6944

The same alphabet in columns shows statistically non-significant differences among treatment means at a 5% probability level, AN24: *Bacillus megaterium*, AN30: *B. aryabhattai*, AN31: *B. megaterium*, and AN35: *B. megaterium.*

The data (Table 1) of root/shoot ratio indicated that the ZSB strains improved rice’s root length/shoot length ratio. All the treatments were effective but with varying degrees of efficacy. The effect of most sole inoculation treatments was non-significant compared to the other treatments but significantly better than that of the un-inoculated control. In co-inoculation treatments, the combined use of *Bacillus megaterium* strains AN24 and AN31 significantly (*p* ≤ 0.05) improved the root/shoot ratio by 6.41% compared to the un-inoculated control. The other co-inoculation *B. megaterium* AN24 with *B. megaterium* AN35, *B. megaterium* AN31 with *B. megaterium* AN35, and *B. megaterium* AN24 with *B. aryabhattai* AN30 showed statistically non-significant results when compared with sole inoculation treatments and un-inoculated control. The ZSB strains significantly improved rice shoot and root dry biomass compared to the un-inoculated control (Table 1). The improvement in shoot dry biomass and root dry biomass due to the sole inoculation ranged from 11.56% to 16.23% and 12.03% to 16.90%, respectively, compared with un-inoculated control. Among sole inoculation treatments, the maximum increase in shoot dry biomass (16.23%) and root dry biomass (16.90%) over the un-inoculated control was obtained from the treatment where the *B. megaterium* AN24 was applied. All the co-inoculation treatments significantly improved the shoot and root dry biomass compared to the un-inoculated control. However, the effect was at par with sole inoculation in most cases. In co-inoculation treatments, the improvement in shoot dry and root dry biomass over the un-inoculated control ranged from 7.55% to 22.72% and 7.24% to 23.76%, respectively. The most effective combination was the *B. megaterium* AN24 with *B. megaterium* AN31, followed by *B. aryabhattai* AN30 with *B. megaterium* AN31.

The data (Table 2) showed that ZSB strains effectively improved the number of tillers plant^–1^. Among the co-inoculation treatments, the combination of *B. megaterium* AN24 and *B. megaterium* AN31 gave the maximum improvement (24.56%) in the number of tillers plant^–1^ compared to the un-inoculated control. In the case of sole inoculation, the *B. megaterium* AN24 showed the maximum improvement (15.79%) in the number of tillers plant^–1^, which was statistically significantly better than the un-inoculated control but at par with other sole inoculation treatments. The results (Table 2) showed that all the sole and co-inoculation treatments of ZSB strains significantly improved the number of grains panicle^–1^ in rice compared with the un-inoculated control. Among co-inoculation treatments, the combination of *B. megaterium* AN24 and *B. megaterium* AN31 gave the best results, with the number of grains panicle^–1^ 14.70 % higher than the un-inoculated control. The sole inoculation was also effective in improving the number of grains panicle^–1^, with results significantly better than the control but all the sole inoculation treatments were statistically at par with each other. The data (Table 2) indicated that the ZSB strains were also significantly effective in improving the 100- grain weight of rice compared to the un-inoculated control. Among the sole inoculation treatments, the *B. megaterium* AN24 showed the best results, with an increase in 100-grain weight over the un-inoculated control group of 17.84%. The results were, however, at par with the sole inoculation of strain *B. megaterium* AN35 and the co-inoculation combination of *B. aryabhattai* AN30 and *B. megaterium* AN35. Among co-inoculation treatments, the combination of *B. megaterium* AN24 and *B. megaterium* AN31 gave the best results, with the 100-grain weight 25.80% higher than the un-inoculated control.

**TABLE 2:** Effect of single and co-inoculation of zinc-solubilizing *Bacillus* strains on yield parameters and yield of rice (*n* = 4).

Treatment	Tillers plant^–1^ (no.)	Grains panicle^–1^ (no.)	100-grain weight (g)	Grain yield (g pot^–1^)	Straw yield (g pot^–1^)
Control	14.2 e	129.3 h	2.10 i	10.4 k	53.2 e
AN24	16.5 a-d	140.8 cd	2.48 cd	12.2 d	61.7 a-d
AN30	15.7 b-e	137.5 d-g	2.33 fg	11.6 g	59.3 cd
AN31	16.0 b-d	138.8 d-f	2.37 ef	11.8 f	60.1 b-d
AN35	16.3 a-d	149.5 de	2.44 de	12.0 e	61.0 a-d
AN24-AN30	15.0 de	135.8 fg	2.26 gh	11.2 i	57.8 de
AN24-AN31	17.7 a	148.3 a	2.65 a	13.0 a	65.1 a
AN24-AN35	15.3 c-e	135.0 g	2.24 h	11.0 j	57.7 de
AN30-AN31	17.3 ab	145.5 ab	2.59 ab	12.7 b	63.9 ab
AN30-AN35	16.7 a-c	143.5 bc	2.53 bc	12.5 c	62.9 a-c
AN31-AN35	15.5 c-e	136.5 e-g	2.28 f-h	11.4 h	58.6 cd
LSD value	1.5285	3.2532	0.00948	0.1159	4.6005

The same alphabet in columns shows statistically non-significant differences among treatment means at a 5% probability level, AN24: *Bacillus megaterium*, AN30: *B. aryabhattai*, AN31: *B. megaterium*, and AN35: *B. megaterium*.

The result (Table 2) showed that rice grain and straw yields improved over the un-inoculated control due to inoculation with ZSB strains. The sole inoculation increased rice grain and straw yield pot^–1^ from 7.91% to 16.03% and 11.47% to 15.91%, respectively, compared to the un- inoculated control. These results were, however, non-significantly different from each other and co-inoculation in the case of straw yield. The increase in grain yield and straw yield due to co-inoculation ranged from 6.34% to 25.26% and 7.47% to 22.23%, respectively, compared with the un-inoculated control. The maximum increase in grain yield and straw yield pot^–1^ was observed due to the combination of *B. megaterium* AN24 and *B. megaterium* AN31, followed by the combination of *B. aryabhattai* AN30 and *B. megaterium* AN31.

### 3.2 Effect of ZSB strains on physiological parameters and antioxidant enzyme activities in rice

The ZSB strains significantly improved physiological traits and antioxidant enzyme activities (Table 3). The co-inoculation of *Bacillus* strains improved the SPAD value from 7.67% to 22.65% and RWC from 9.19% to 28.03%. The maximum improvement in SPAD value (28.7 ± 0.41 to 35.2 ± 0.32) was observed due to the co-inoculation combination of *B. megaterium* AN24 and AN31 strains. The sole inoculation of *Bacillus* strains also improved the SPAD value (10.37%–16.73%) and relative water content (13.13%–20.02%) over the un-inoculated control. The *B. megaterium* AN24 was the most effective among the sole inoculation treatments in improving rice leaves’ SPAD value and relative water content.

**TABLE 3:** Effect of single and co-inoculation of zinc-solubilizing *Bacillus* strains on rice physiology and antioxidant enzyme activities (*n* = 4).

Treatment	SPAD value	Relative water content (%)	Superoxide dismutase activity (U mg^–1^ FW)	Peroxidase activity (U mg^–1^ FW)	Catalase activity (U mg^–1^ FW)	Ascorbate peroxidases activity (U mg^–1^ FW)
Control	28.7 h	57.7 j	8.1 h	21.5 h	5.80 j	2.30 k
AN24	33.3 cd	69.3 d	12.9 bc	25.1 cd	6.31 cd	4.21 d
AN30	31.7 e-g	65.3 fg	11.4 de	23.5 fg	6.17 fg	3.57 g
AN31	32.3 d-f	66.4 f	11.9 d	24.0 ef	6.22 ef	3.83 f
AN35	32.7 de	68.1 e	12.3 cd	24.7 de	6.27 de	4.06 e
AN24-AN30	31.1 g	63.5 hi	10.1 fg	23.1 g	6.05 hi	3.08 i
AN24-AN31	35.2 a	73.9 a	15.4 a	27.6 a	6.48 a	4.91 a
AN24-AN35	30.9 g	63.0 i	9.5 g	22.8 g	5.98 i	2.83 j
AN30-AN31	34.5 ab	72.6 b	14.5 a	26.7 b	6.42 ab	4.68 b
AN30-AN35	33.9 bc	70.6 c	13.4 b	25.8 c	6.36 bc	4.43 c
AN31-AN35	31.4 fg	64.6 gh	10.8 ef	23.4 fg	6.12 gh	3.31 h
LSD value	1.0705	1.1591	0.9326	0.8294	0.0746	0.0779

The same alphabet in columns shows statistically non-significant differences among treatment means at a 5% probability level, AN24: *Bacillus megaterium*, AN30: *B. aryabhattai*, AN31: *B. megaterium*, and AN35: *B. megaterium*.

The results (Table 3) showed that ZSB strains effectively improved the superoxide dismutase activity in rice leaves. Among the co-inoculation treatments, the combination of *B. megaterium* AN24 and *B. megaterium* AN31 gave the best results in the case of superoxide dismutase activity compared to the un-inoculated control and other treatments. In the case of sole inoculation, the *B. megaterium* AN24 showed the maximum improvement in superoxide dismutase activity, which was statistically significantly better than the un-inoculated control. The sole and co-inoculation treatments of ZSB strains also significantly improved the peroxidase activity in rice leaves compared with the un-inoculated control (Table 3). Among co-inoculation treatments, the combination of *B. megaterium* AN24 and *B. megaterium* AN31 gave the best results, with the peroxidase activity 28.79% higher than the un- inoculated control. The sole inoculation also improved the peroxidase activity, with results significantly better than those of the control.

The data (Table 3) indicated that the ZSB strains were also significantly effective in improving the catalase activity and ascorbate peroxidase activity in rice leaves compared to the un- inoculated control. Among the sole inoculation treatments, the *B. megaterium* AN24 showed the best results, with an increase of 8.80% in catalase activity and 83.07% in ascorbate peroxidase activity over the un-inoculated control. The combination of *B. megaterium* AN24 and *B. megaterium* AN31 among co-inoculation treatments gave the best catalase and ascorbate peroxidase activity in U mg^–1^ FW. The results were statistically at par with the combination *B. aryabhattai* AN30 and *B. megaterium* AN31 but significantly better than the other combinations and the un-inoculated control.

### 3.3 Effect of ZSB strains on mineral nutrients shoot, root, and rice grains

**FIGURE 2:**
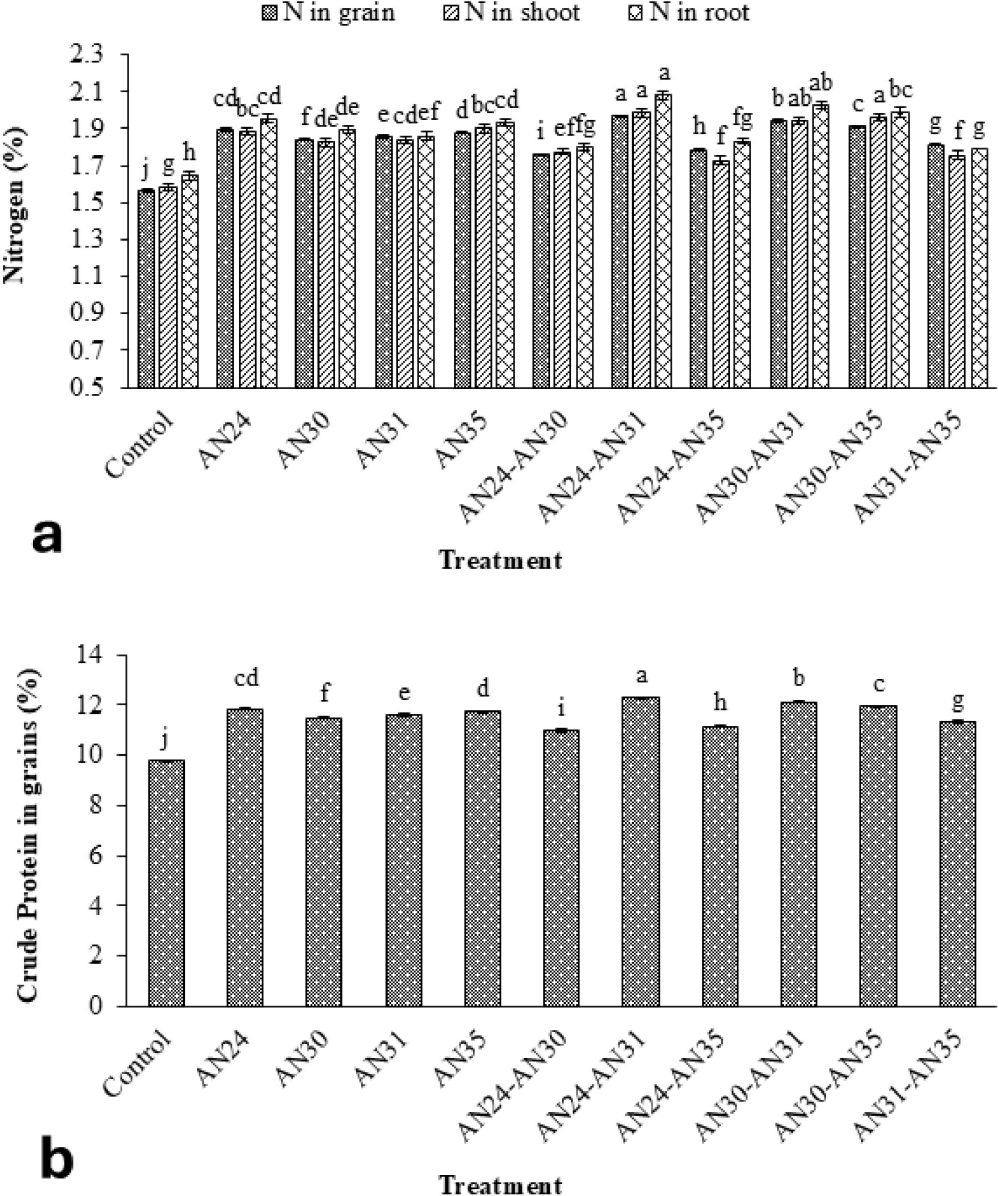
Efficacy of single and co-inoculation of zinc-solubilizing *Bacillus* strains in improving **(a)** nitrogen contents in shoot, root, and grains of rice and **(b)** crude protein in pot experiment (*n* = 4). Bars of treatments with similar letters are statistically at par. Where, AN24: *Bacillus megaterium*, AN30: *B. aryabhattai*, AN31: *B. megaterium*, and AN35: *B. megaterium.*

The ZSB strains significantly improved N concentration in rice plants’ grains, straw, and roots compared to the un-inoculated control (Figure 2a). The improvement in N concentration in grains, straw, and roots due to the co-inoculation of *Bacillus* strains ranged from 12.40% to 25.82%, 8.99% to 25.24%, and 8.66% to 26.60%, respectively, compared with the un- inoculated control. The most effective combination was the *B. megaterium* AN24 with *B. megaterium* AN31, followed by *B. aryabhattai* AN30 with *B. megaterium* AN31. Among sole inoculation treatments, the maximum increase in N concentration grains (21.12%) and roots (18.69%) over the un-inoculated control was obtained from the treatment where the *B. megaterium* AN24 was applied. However, in the case of straw N concentration, *B. megaterium* AN35 showed the best results. Regarding crude protein concentration in rice grains, all the inoculated and co-inoculation treatments of ZSB strains demonstrated results like those of N concentration in rice grains (Figure 2b).

**FIGURE 3:**
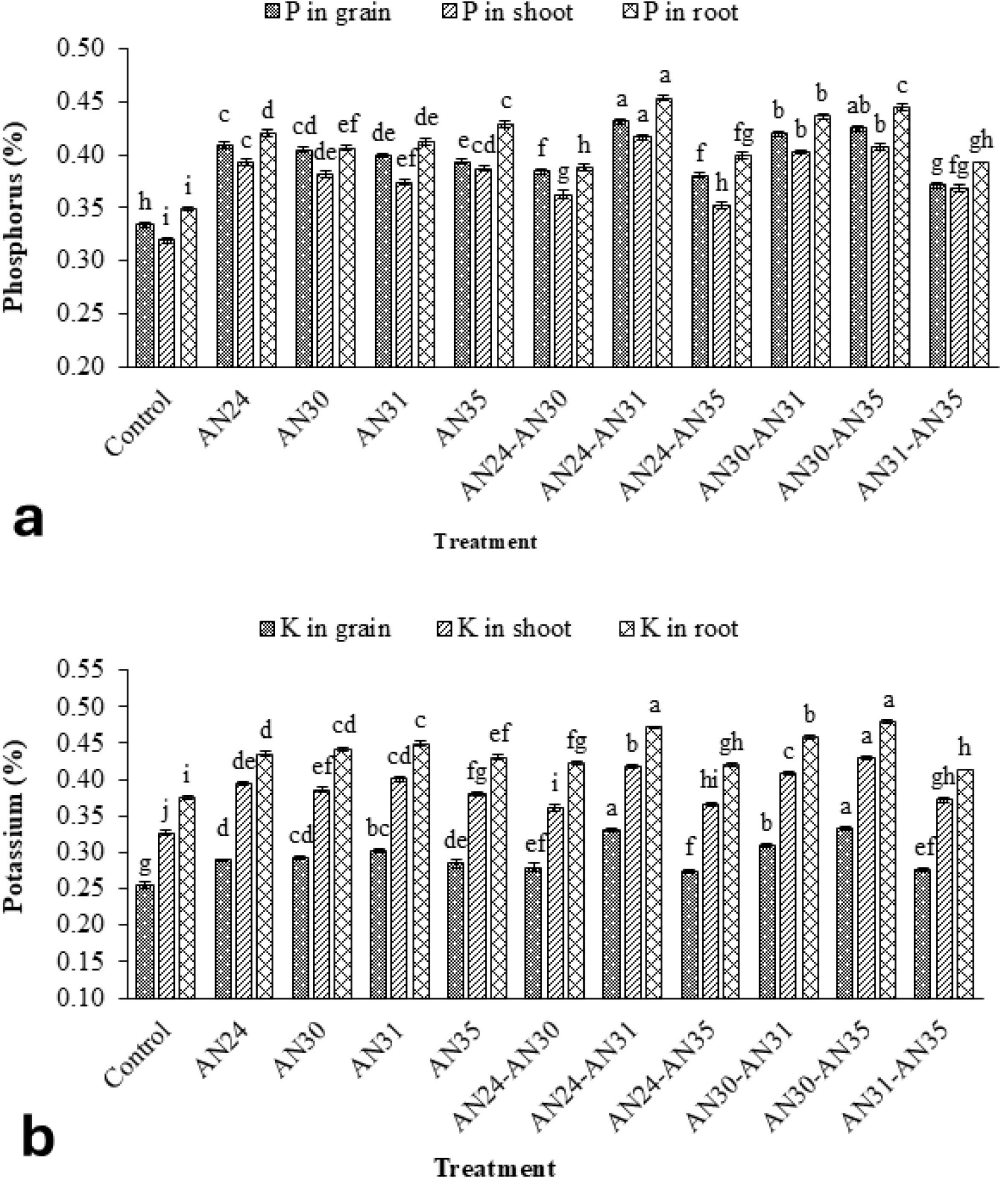
Efficacy of single and co-inoculation of zinc-solubilizing *Bacillus* strains in improving **(a)** phosphorus and **(b)** potassium contents in shoot, root, and grains of rice in pot experiment (*n* = 4). Bars of treatments with similar letters are statistically at par. Where, AN24: *Bacillus megaterium*, AN30: *B. aryabhattai*, AN31: *B. megaterium*, and AN35: *B. megaterium.*

The data (Figure 3a) showed that the ZSB strains significantly improved P concentration in rice grains, straw, and roots compared to the un-inoculated control. The co- inoculation combinations improved the P concentration in rice grains from 8.74% to 30.11%, straw P contraction from 7.40% to 28.43%, and P concentration in roots from 7.58% to 28.19%. The maximum improvement in P concentration in rice grains, straw, and roots compared to the un-inoculated control was observed due to the co- inoculation combination of *B. megaterium* AN24 and *B. megaterium* AN31. The sole inoculation of *Bacillus* strains also significantly improved the P concentration in rice grains, straw, and roots over the un-inoculated control. The results (Figure 3b) indicated that the ZSB strains were also significantly effective in improving the K concentration of rice grains, straw, and roots compared to the un- inoculated control. Among the sole inoculation treatments, the *B. megaterium* AN31 showed the best results, with an increase of 22.51%, 22.65%, and 19.87% in K concentration of rice grains, straw, and roots, respectively, over the un-inoculated control. Among co- inoculation treatments, the combination of *B. megaterium* AN24 and AN31 gave the best results, with an increase of 27.09%, and 25.73% in K concentration of rice grains and roots, respectively, over the un-inoculated control. The results were statistically at par with the combination of *B. aryabhattai* AN30 and *B. megaterium* AN31, which also gave the best results for K concentration in rice straw, where the increase was 31.37% over the un-inoculated control.

**FIGURE 4:**
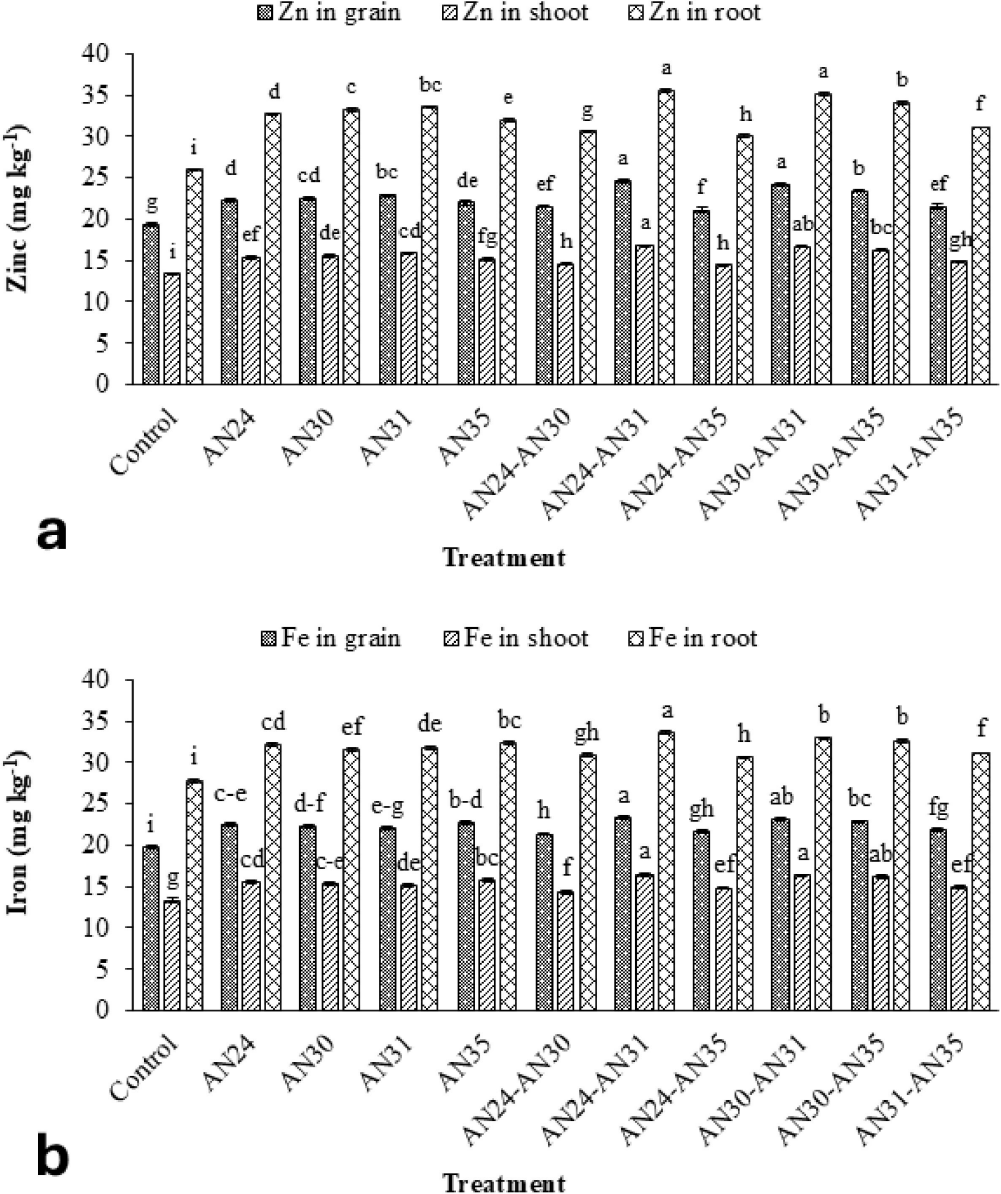
Efficacy of single and co-inoculation of zinc-solubilizing *Bacillus* strains in improving **(a)** zinc and **(b)** iron contents in shoot, root, and grains of rice in pot experiment (*n* = 4). Bars of treatments with similar letters are statistically at par. Where, AN24: *Bacillus megaterium*, AN30: *B. aryabhattai*, AN31: *B. megaterium*, and AN35: *B. megaterium.*

The data (Figure 4a) showed that the ZSB strains significantly improved zinc concentration in rice grains, straw, and roots compared to the un-inoculated control. The co-inoculation improved the zinc concentration in rice grains from 9.03% to 26.84%, straw zinc concentration from 7.85% to 25.42%, and zinc concentration in roots from 15.40% to 36.67%. The maximum improvement in zinc concentration in rice grains, straw, and roots compared to the un-inoculated control was observed due to the co-inoculation combination of *B. megaterium* AN24 and *B. megaterium* AN31. The sole inoculation of *Bacillus* strains also significantly improved the zinc concentration in rice grains, straw, and roots over the un-inoculated control.

The ZSB strains significantly improved iron concentration in rice plants’ grains, straw, and roots compared to the un-inoculated control (Figure 4b). The improvement in iron concentration in grains, straw, and roots due to the co-inoculation ranged from 9.03% to 26.84%, 7.87% to 23.22%, and 9.87% to 20.56%, respectively, compared with the un- inoculated control. The most effective combination was the *B. megaterium* AN24 with *B. megaterium* AN31, followed by *B. aryabhattai* AN30 with *B. megaterium* AN31. Among sole inoculation treatments, the maximum increase in iron concentration grains (15.10%), straw (17.98%), and roots (16.34%) over the un-inoculated control was obtained from the treatment where the *B. megaterium* AN35 was applied.

### 3.4 Effect of ZSB strains on mineral contents and bacterial population in soil under rice

The results (Table 4) showed that the total N concentration in soil under rice was significantly improved due to inoculation with ZSB strains compared to the un-inoculated control. The improvement in total N concentration in soil due to the co-inoculation ranged from 15.58% to 28.42% compared with the un-inoculated control. The most effective combination was the *B. megaterium* AN24 with *B. megaterium* AN31, followed by *B. aryabhattai* AN30 with *B. megaterium* AN31. Among sole inoculation treatments, the maximum increase in total N concentration soil after rice harvest (24.14%) over the un- inoculated control was obtained from the treatment where the *B. megaterium* AN24 was applied. The data showed that the ZSB strains significantly improved the available phosphorous content in the soil compared to the un-inoculated control (Table 4). The co-inoculation combinations improved the available phosphorus concentration in the soil after rice harvest, ranging from 18.05% to 30.73%. The maximum improvement in available phosphorus concentration in the soil compared to the un-inoculated control was observed due to the co- inoculation combination of *B. megaterium* AN24 and *B. megaterium* AN31. The sole inoculation of *Bacillus* strains also significantly improved the available phosphorus concentration in the soil after rice harvest over the un-inoculated control.

**TABLE 4:** Effect of single and co-inoculation of zinc-solubilizing *Bacillus* strains on soil mineral contents and bacterial population under rice cultivation (*n* = 4).

Treatment	Total *N* (%)	Available P (mg kg^–1^)	Extractable K (mg kg^–1^)	Iron (mg kg^–1^)	Zinc (mg kg^–1^)	Bacterial population (×10^4^ cfu/ g)
Control	0.0213 g	10.9 h	58.0 h	4.57 i	0.69 h	31.5 f
AN24	0.0261 a-d	14.0 bc	70.0 def	4.99 de	0.77 cd	38.0 bc
AN30	0.0255 de	13.9 cd	71.5 c-e	5.03 d	0.77 cd	36.0 cd
AN31	0.0257 c-e	13.7 d	72.5 b-d	5.08 c	0.78 bc	36.0 cd
AN35	0.0259 b-d	13.4 e	68.7 e-g	4.94 e	0.76 de	37.0 bc
AN24-AN30	0.0243 f	13.2 ef	66.8 fg	4.78 h	0.73 fg	33.3 ef
AN24-AN31	0.0270 a	14.3 a	76.0 a	5.26 a	0.81 a	41.5 a
AN24-AN35	0.0239 f	13.1 fg	65.7 g	4.83 g	0.72 g	33.5 ef
AN30-AN31	0.0268 ab	14.1 ab	75.0 ab	5.22 a	0.80 a	40.5 a
AN30-AN35	0.0266 a-c	14.2 a	73.7 a-c	5.16 b	0.79 ab	39.3 ab
AN31-AN35	0.0248 ef	12.9 g	68.0 fg	4.88 f	0.74 ef	34.5 de
LSD value	0.0010	0.2234	3.3692	0.0464	0.0151	2.3021

The same alphabet in columns shows statistically non-significant differences among treatment means at a 5% probability level, AN24: *Bacillus megaterium*, AN30: *B. aryabhattai*, AN31: *B. megaterium*, and AN35: *B. megaterium*.

The results (Table 4) indicated that the extractable K in the soil after rice harvest was significantly improved by the ZSB strains compared to the un-inoculated control. Among the sole inoculation treatments, the *B. megaterium* AN31 showed the best results, with an increase of 25% in extractable K concentration in the soil after rice harvest over the un- inoculated control. Among co-inoculation treatments, the combination of *B. megaterium* AN24 and *B. megaterium* AN31 gave the best results, with an increase of 31.03% in extractable K in the soil over the un-inoculated control. The results were statistically at par with the combination of *B. aryabhattai* AN30 and *B. megaterium* AN31 and *B. aryabhattai* AN30 and *B. megaterium* AN35.

The ZSB strains significantly improved iron concentration in the soil after rice harvest compared to the un-inoculated control (Table 4). The improvement in iron concentration in soil due to the co-inoculation ranged from 4.63% to 15.09% compared with the un- inoculated control. The most effective combination was the *B. megaterium* AN24 with *B. megaterium* AN31, followed by *B. aryabhattai* AN30 with *B. megaterium* AN31. Among sole inoculation treatments, the maximum increase in iron concentration in soil (11.12%) over the un-inoculated control was obtained from the treatment where the *B. megaterium* AN31 was applied. The data (Table 4) showed that the ZSB strains significantly improved zinc concentration in soil under rice compared to the un-inoculated control. The co-inoculation improved the zinc concentration (0.72–0.81 ppm) in the soil after rice harvest from 4.27% to 16.04% as compared to un-inoculated control (0.69 ppm). The maximum improvement in zinc concentration in soil compared to the un- inoculated control was observed due to the co-inoculation combination of *B. megaterium* AN24 and *B. megaterium* AN31. The results were statistically at par with the combination of *B. aryabhattai* AN30 and *B. megaterium* AN31 and *B. aryabhattai* AN30 and *B. megaterium* AN35 but significantly better than control. The sole inoculation of *Bacillus* strains also significantly improved the zinc concentration in soil under rice over the un-inoculated control.

The results (Table 4) revealed that ZSB strains significantly improved the soil bacterial population in the rice rhizosphere compared to the un-inoculated control. Among co-inoculation treatments, the combination of *B. megaterium* AN24 and *B. megaterium* AN31 gave the best results regarding the soil bacterial population in the rice rhizosphere compared with the un- inoculated control. The results were statistically at par with the combination of *B. aryabhattai* AN30 and *B. megaterium* AN31 and *B. aryabhattai* AN30 and *B. megaterium* AN35 but significantly different from the un-inoculated control. The sole inoculation of *Bacillus* strains also significantly improved the soil bacterial population in the rice rhizosphere over the un- inoculated control. Among sole inoculation treatments, the *Bacillus megaterium* AN24 gave the maximum improvement (20.63%) in the soil bacterial population compared to the un- inoculated control, which was statistically at par with other sole inoculation treatments but significantly better than the control.

### 3.5 Correlation and principal component analyses

**FIGURE 5:**
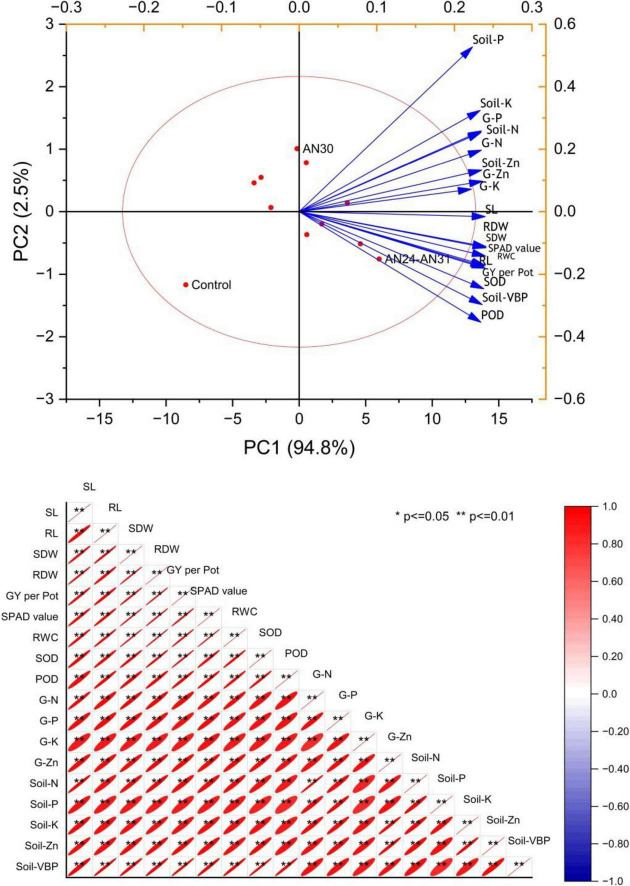
Multivariate analysis of the critical growth, physiological and yield parameters of rice *viz*. Biplot of principal component analysis (PCA) and Pearson’s correlation analysis.

The PCA biplot (Figure 5) illustrates that ZSB strains had a significant influence on rice growth, yield, physiological traits, soil mineral content, and viable bacterial population (VBP). Principal components PC1 and PC2, with eigenvalues 17.07 and 0.454, explaining 94.8% and 2.5% of the total variance, respectively. The parameters shoot length and root length show the highest loadings 0.23983 and 0.23819, respectively. On the other hand, treatments, particularly AN24+AN31 and AN30+AN31, exhibited strong positive scores 6.01548 and 0.45869, respectively, for PC1, indicating their substantial impact on rice growth, physiology and yield. Sole inoculations showed moderate effects. Pearson’s correlation analysis confirmed strong positive relationships among different rice parameters like plant growth, yield, physiology, soil nutrients, and VBP.

## 4 Discussion

Food security remains a major global challenge, especially under the current climate change scenario. According to the UN, the global population has risen from 7 billion in 2010 to 8.1 billion in 2024 and is projected to exceed 9.7 billion by 2050. This rapid growth places immense pressure on food systems (Rahut et al., 2022). While food availability has improved, many people, particularly in developing countries, still lack access to nutritious food. Among micronutrient deficiencies, zinc (Zn) deficiency in staple grains poses a significant health risk, affecting millions worldwide (Kiran et al., 2022). Zinc solubilizing bacteria have been well documented to solubilize insoluble Zn compounds in soils by adopting any of the mechanisms: proton extrusion, redox reactions, siderophores, exopolysaccharides (EPS) and organic acids production (Mumtaz et al., 2017). These bacteria possess multifarious plant growth-promoting (PGP) traits, including zinc solubilization can be used to improve crop productivity and nutrient quality and is an eco-friendly, low-cost, and sustainable approach (Yadav et al., 2022; Singh et al., 2024). Based on the facts, the present study explored the potential of ZSB strains to enhance rice growth, physiology, yield, and grain quality.

### 4.1 Effect of *Bacillus* strains on growth, yield, and physiological parameters of rice

In the current study, inoculation/co-inoculation with *Bacillus* strains significantly increased the plant height, root length, root/shoot ratio, shoot dry biomass, root dry biomass, number of tillers plant^–1^, flag leaf length, panicle length, number of grains panicle^–1^, panicle weight, 100-grain weight, grain yield, and straw yield of rice as compared to un-inoculated control. The results regarding growth improvement are in line with the findings of Gontia-Mishra et al. (2017), which described the production of solubilizing substances (organic acids and enzymes), EPS and siderophore production responsible for growth improvement. However, the results regarding yield attributes are in line with the findings of Shakeel et al. (2024), which described the improvement as a result of balanced nutrient uptake in the rice plant through solubilization of entrapped nutrients by the Zn-solubilizing bacteria and nutrients translocation in the higher parts. Zinc solubilizing *Bacillus* strains also significantly improved the physiological parameters, including relative water content, SPAD value, POD activity, SOD activity, APX activity, and catalase activity. Our results regarding antioxidant status are in line with the findings of Alharbi et al. (2022). It has been reported many times that the *Bacillus* strains are a good candidate as plant growth promoting rhizobacteria (PGPR) and significantly improve plant growth and development (Porcel et al., 2014; Ahmad et al., 2021). They do so by production of several phytohormones like auxins, gibberellins and cytokinins (Mumtaz et al., 2017; Nazli et al., 2020, 2021; Saeed et al., 2023; Ahmad et al., 2023a), regulate soil exoenzyme activities like phosphate esterase and ß-D glucosidase (Iqbal et al., 2024), which help in improving minerals solubilization (especially P and Zn), improvement in plant physiological processes like chlorophyll contents, photosynthesis, and respiration (Naseer et al., 2020; Singh et al., 2021; Nadeem et al., 2021), induction of tolerance against various stresses (salinity, heat, drought, pests and diseases) (Khan et al., 2020; Malik et al., 2024), and improve antioxidant enzyme activities (El-Beltagi et al., 2022b) and reduce the oxidative damage.

The strains used in the study possess multifarious plant growth-promoting traits, including zinc solubilization, siderophores production, hydrogen cyanide (HCN) production, and organic acids production (Naseer et al., 2020). These traits might have helped plants perform better than un-inoculated rice plants. Moreover, the co-inoculation of these strains might have worked synergistically to upregulate plant growth and physiological functions; thus, co-inoculated plants showed better growth, yield, and physiological parameters as compared to the sole inoculation of these strains (Ahmad et al., 2013; Khan et al., 2020).

### 4.2 Effect of *Bacillus* strains on mineral contents of rice grain, straw, and roots

Soil mineral nutrients play a crucial role in determining plant growth and productivity, especially under heterogeneous soil conditions. Soil of arid and semi-arid regions are deficient in essential plant nutrients, e.g., a deficiency of potassium and phosphorus may induce N deficiency in these soils (Ayangbenro and Babalola, 2021). Applying *Bacillus* inoculants in the present study significantly improved crude protein contents in rice grains up to 27% with improved N, P, K, Fe and Zn contents up to 26%, 30%, 29%, 19%, and 27%, respectively. The results of nutrients translocation from roots to upper portion are lower than Singh et al. (2023) and similar to Yadav et al. (2023). Shi et al. (2024) emphasized that this nutrients improvement in the rice gains is attributed to the inoculation and co-inoculation of *Bacillus* strains by enhancing FUE in nutrient-deficient soils. *Bacillus* strains can enhance mineral solubilization through organic acids, siderophores and EPS production (Mumtaz et al., 2017; Anwar et al., 2025), by mineralization and solubilization of nutrients in soils (Naseer et al., 2020; Ahmad et al., 2023b). Another aspect of higher mineral uptake might be the auxin-induced higher root proliferation (Mumtaz et al., 2017; Saeed et al., 2023), which might enhance the area of root water and minerals absorption by modifying the physiological processes in plants (Singh et al., 2021; Nadeem et al., 2021). Ghazanfar et al. (2024) and Murad et al. (2024) also described similar mechanisms for enhancement of nutrient availability and uptake in rice as inoculated plants performed better than un-inoculated control plants. In addition, the combined use of *Bacillus* strains performs better than the sole use of these strains (Murad et al., 2024; Perveen et al., 2023), which might be due to the upregulation of plant growth and physiological functions owing to synergism.

### 4.3 Effect of *Bacillus* strains on the health and biology of soil under rice

Inoculation with the *Bacillus* strains in the current study significantly enhanced the nutritional status of the studied soil by providing higher available concentrations of N, P, K, Zn, and Fe in the rice rhizosphere. Moreover, co-inoculation was also more efficient in improving the soil nutrient status and biology (bacterial population) of soil under rice than sole inoculation. The enhancement of fertility and biological properties are in line with the findings of Ng et al. (2022) which describes that the application of *Bacillus* and *Pseudomonas* sp. has the potential to enhance soil fertility and health. These bacteria have been well-documented to mineralize complex organic compounds in soil and their ability to solubilize insoluble mineral complexes (Kumari et al., 2016; Shabaan et al., 2022; Mumtaz et al., 2017). The nutritional status of the soil might be enhanced by the applied strains by zinc and phosphorus solubilization through organic acids and possess siderophores production ability proved in our previous study Naseer et al. (2020). These bacteria use different processes to solubilize the insoluble Zn compounds *viz.*, proton extrusion, redox reactions, siderophores, exopolysaccharides (EPS) and organic acids production. The more studied mechanism is the production of low molecular weight organic acids (Mumtaz et al., 2017), which reduce pH in the microenvironment, thus inducing a favorable environment for nutrients solubility (Fe, Zn, P) and availability (N, K) to plants. Moreover, the enhancement in microbial populations is attributed to the inoculation and co-inoculation of the *Bacillus* stains in the soil, which helps to not only solubilize the insoluble minerals and make the rhizosphere a nutrient-rich zone for bacterial populations to flourish (Du et al., 2022). It has been reported that inoculation with microbial inoculants positively influences the soil microbial population by improving soil nutrient status and biological properties (Rizvi et al., 2022; Iqbal et al., 2024).

Afterall the study has limited scope to only rice crop, and under arid climatic conditions and sandy loam soil. Therefore, the best performing combination must be tested for other cereals (wheat and maize), under fields of different climatic zones and different soils for development of a biofertilizer for sustainable production of cereals.

## 5 Conclusion

The results of this study demonstrate that inoculation with zinc-solubilizing *Bacillus* (ZSB) strains significantly enhances rice growth, yield, physiological functions, and nutrient uptake, particularly under nutrient-deficient soil conditions. Notably, co-inoculation proved more effective than individual applications, with the combination of *Bacillus megaterium* strains AN24 and AN31 showing the most pronounced improvements in plant performance and soil biological properties.

These findings highlight the potential of microbial co-inoculants as practical, eco-friendly biofertilizers for sustainable rice production. Adoption of such formulations could reduce dependency on synthetic fertilizers, improve soil fertility, and contribute to long-term agricultural resilience, especially in nutrient-poor or degraded soils. To translate these findings into real-world impact, further validation through large-scale, on-farm trials is essential. Future research should also explore the long-term agronomic and economic benefits, potential scalability, and integration into existing fertilization regimes, along with molecular identification of the nutrient solubilizer genes in the studied bacterial strains. Policymakers and extension services are encouraged to support the development, registration, and dissemination of such biofertilizer technologies. Promoting these microbial solutions through farmer awareness programs and subsidy frameworks can play a critical role in enhancing food security, improving grain nutritional quality, and supporting sustainable agricultural practices.

## Data Availability

The datasets presented in this study can be found in online repositories. The names of the repository/repositories and accession number(s) can be found here: https://www.ncbi.nlm.nih.gov/genbank/, accession numbers MN005926, MN005927, MN005928, and MN005929.
